# Development and Evaluation of a Caregiver Reported Quality of Life Assessment Instrument in Dogs With Intracranial Disease

**DOI:** 10.3389/fvets.2020.00537

**Published:** 2020-08-18

**Authors:** Rebecca Weiske, Maureen Sroufe, Mindy Quigley, Theresa Pancotto, Stephen Werre, John H. Rossmeisl

**Affiliations:** ^1^Department of Small Animal Clinical Sciences and Veterinary and Comparative Neuro-Oncology Laboratory, Department of Small Animal Clinical Sciences, Virginia-Maryland College of Veterinary Medicine, Virginia Tech, Blacksburg, VA, United States; ^2^The Study Design and Statistical Analysis Laboratory, Department of Small Animal Clinical Sciences, Virginia-Maryland College of Veterinary Medicine, Virginia Tech, Blacksburg, VA, United States

**Keywords:** animals, brain, canine, health status, quality of life, surveys and questionnaires, welfare

## Abstract

In veterinary medicine, quality of life (QOL) assessment instruments, which are important components of the holistic evaluation of treatment success, have largely not included organ-specific concerns that may be broadly relevant to caregivers of dogs with intracranial disease. The objective of this study was to identify core questionnaire items and domains that contribute to health-related QOL (HRQOL) in dogs with intracranial disease. A questionnaire was developed that contained 39 QOL-related items encompassing physical, social/companionship, and brain-specific domains associated with the treatment of dogs with intracranial disease, and administered to caregivers of 56 dogs diagnosed with genetic, inflammatory, neoplastic, traumatic, and vascular brain diseases, 52 healthy dogs, and 20 dogs with non-neurological illnesses. Clinician derived functional measures of each dog's health status including chronic pain, Karnofsky performance, and modified Glasgow coma scale scores were also recorded. Principal component analysis refined the final questionnaire, termed the CanBrainQOL-24, to 24-items within the three domains with a minimum Cronbach's alpha of 0.7, indicative of good internal consistency. The CanBrainQOL-24 discriminated between healthy and diseased dogs. Physical and brain-specific domains were significantly different between dogs with intracranial and non-neurological diseases. Significant correlations were observed between owner reported visual analog scores and CanBrainQOL-24 scores, as well between clinician derived functional status measures and owner reported QOL. The CanBrainQOL-24 contains core questions relevant to caregiver assessment of HRQOL in dogs with a variety of intracranial diseases, and provides information that is complementary to clinician derived functional outcome measures.

## Introduction

Health related quality of life (HRQOL) is a multidimensional concept that considers quality of life (QOL) in the context of health and disease. The World Health Organization defines HRQOL in domains related to physical, social, and neurobehavioral well-being as perceived by the patient (1). HRQOL assessments can play a significant role in clinical decision-making, as well as determining the effectiveness of treatments in numerous human diseases (2). In veterinary medicine, there is a growing recognition of the need to incorporate patient-centered outcomes along with traditional objective health outcomes into practice, and to focus on the patients' and caregivers' needs and experiences to define goals for and expectations of treatment (3, 4). There is further evidence that an intervention can be perceived by a human patient or animal caregiver as meaningful if it improves a patient's subjective well-being, even if it does not significantly alter commonly used objective outcome measures, such as overall survival (5, 6).

Several general and disease specific HRQOL assessment tools have been developed for use in dogs (7–11), including questionnaires tailored for evaluating canine neurological diseases such as idiopathic/genetic epilepsy (IE) and spinal cord injury (12–14). These tools focus on the QOL consequences of the dog's health status and have been found to provide information that is complementary to traditional objective measures of health that can be useful to clinicians and caregivers. While the physical and behavioral effects of intracranial diseases on dogs are well recognized, the impact of these on HRQOL have not been thoroughly investigated, and this may be in part be attributed to the current lack of HRQOL tools developed specifically to evaluate dogs with structural intracranial diseases or structural epilepsy (12, 14, 15). Our previous experience with a generic HRQOL questionnaire in dogs with brain tumors suggested that existing general HRQOL instruments may not be sensitive to some problems that are unique to and common in animals with brain disorders (16). Thus, there is an unmet need to assess the impact of intracranial disease and its treatment on caregiver-reported HRQOL in dogs. Incorporation of HRQOL outcomes into clinical management practices is particularly relevant to patients with intracranial disease, where evidence-based care requires increasingly complex, often invasive, and expensive interventions.

The objective of this study was to identify core questions and domains that contribute to HRQOL in dogs with intracranial disease. It was hypothesized that a HRQOL instrument that incorporated brain-specific items could discriminate between healthy dogs and those with intracranial disease, as well as dogs with intracranial disease from clinically ill dogs with non-neurological illnesses.

## Methods

### Questionnaire Development and Design

**Figure 1:**
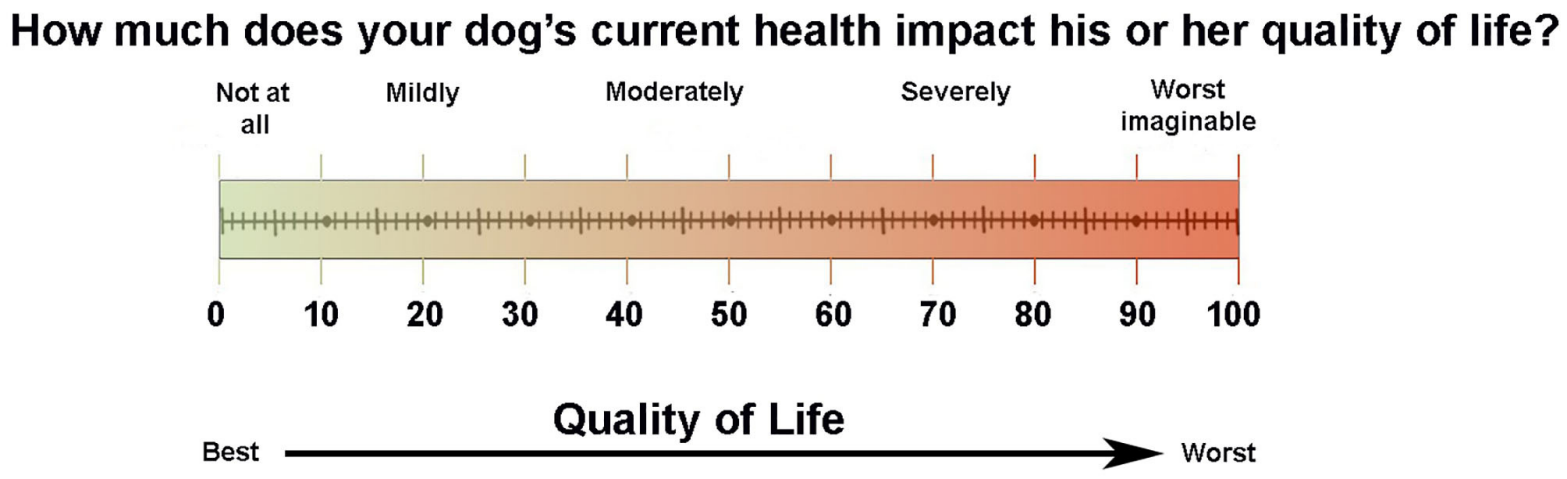
Visual analog quality of life scale.

A pilot questionnaire was designed to include HRQOL items considered relevant to the owners or caregivers of dogs diagnosed with and undergoing treatment for intracranial diseases. The questionnaire was comprised of three sections, the first of which consisted of 12 close-ended queries requesting patient identifying and medical data such as the signalment, diagnosis, duration of neurological illness, type of treatment(s) received, and other concurrent diseases and therapies. The second section included 84 close-ended question items that targeted specific HRQOL areas potentially impacted in dogs with intracranial disease. Items included in the second section were created using input from board-certified veterinarians with experience managing dogs with intracranial disease, or derived from published HRQOL instruments for dogs (7, 11) and humans (1, 17). All items in the second section were structured using a Likert-type interval (1–5) rating scale. The third section of the questionnaire asked the respondent to rate their dog's overall QOL using a visual analog scale (VAS; Figure 1) and answer 2 close-ended (Yes/No) questions: (1) if there were any other health-related items that impacted their dog that were not represented in the survey, and (2) if any responses to survey items were attributable to treatment(s) administered to their dog. If respondents answered “yes” to any of the closed-ended questions, they were prompted to provide an open-ended comment as to the type (what) and severity (how bothersome) of their observations.

The pilot questionnaire was initially evaluated for content validity by a focus group consisting of eight informed veterinary subject matter experts (board-certified veterinary specialists, *n* = 2; veterinarians in general practice, *n* = 2; licensed veterinary nurses, *n* = 2; epidemiologist, *n* = 1; and animal behaviorist, *n* = 1). The face validity of the questionnaire was assessed by administering the survey by telephone to the owners of 10 dogs diagnosed with intracranial disease (brain tumor, *n* = 3; meningoencephalitis, *n* = 3; ischemic stroke, *n* = 2; and IE, *n* = 2). Owners and members of the focus group were informed as to the objective of the questionnaire, but QOL was not specifically defined.

Owner and expert rater feedback about item relevance and readability guided modification and construction of the CanBrainQOL-39 questionnaire. The CanBrainQOL-39 questionnaire also consisted of three sections, with the first patient medical data section being unmodified from the pilot evaluation, the second section consisting of 39 HRQOL related items (Table S1), and a third section asking the respondent to rate their dog's overall QOL using the VAS and answer 2 close-ended (Yes/No) questions: (1) if there were any other QOL items that impacted their dog that were not represented in the survey, and (2) if any responses to survey items were attributable to treatment(s) administered to their dog. If respondents answered “yes” to any of the closed-ended questions, they were prompted (but not required) to provide an open-ended comment as to the type (what) and severity (how bothersome) of their observations. Total possible HRQOL scores for the second section ranged from 39-195. For both the second section questionnaire total HRQOL and the VAS section, higher scores indicate worse QOL.

### Questionnaire Domain Classification

Items in the questionnaire were assigned to three domains: physical well-being, social well-being and companionship, and organ (brain) specific functional and cognitive domains of the dog, based on criteria from prior veterinary and human research (1, 7, 8). Items 1–22 were considered to fall within the physical domain, items 23–30 the social and companionship domain, and items 31–39 the brain-specific domain, as assessed by the caregiver.

### Recruitment of Respondents

The medical records database of the authors' institution was searched for dogs ≥ 1 year of age diagnosed with the following intracranial diseases: congenital hydrocephalus (18), IE/genetic epilepsy [Tier II confidence level (19)], ischemic infarction (20), meningoencephalitis of unknown etiology [MUE (21)], brain tumor (22), or traumatic brain injury [TBI (23)] that were previously discharged from the hospital. For inclusion, it was required that the etiologic diagnosis in each dog was established by a board-certified neurologist according to published disease-specific criteria based on results of a neurological examination, clinicopathological testing, a magnetic resonance imaging examination of the brain, analyses of cerebrospinal fluid, or histopathologic examination of brain tissue (18–23).

Records meeting these criteria were cross-referenced with the clinical appointment schedule of the neurology and neurosurgery service to identify caregivers who had made a future appointment for continuing care of their dog's intracranial disorder. These caregivers were contacted by telephone or electronic mail to inquire if they would be willing to complete the CanBrainQOL-39 questionnaire as a part of their dog's future appointment. Those responding affirmatively were forwarded a secure electronic link to an online version of the questionnaire one week prior to their scheduled appointment. Caregivers completing the questionnaire were required to be ≥ 18 years of age and have lived with their dog for at least 6 months preceding the diagnosis of intracranial disease. Caregivers were instructed to complete the questionnaire in the context of the degree to which clinical signs or treatment of intracranial disease affected their dog's QOL for the 7 days preceding their completion of the survey. The online questionnaire was constructed such that it was required for respondents to answer each multiple choice item in order to advance to the next question. It was possible for owners to skip questionnaire items if they were completing the written version of the survey. At the time of each dog's appointment, the attending clinician also recorded canine chronic pain (CPS), Karnofksy performance (KPS), and modified Glasgow coma (MGCS) scale scores, using previously described methods (22–24). All diseased dogs included in this study were receiving treatment for their disease at the time surveys were administered.

Healthy dogs were recruited from the population presenting for preventative health care visits or dental prophylaxis to the outpatient medicine or community practice services. Dogs were considered healthy based on a lack of significant abnormalities on physical and neurological examinations performed by attending faculty veterinarians, and complete blood count and serum biochemical profile results that were within reference ranges. An additional cohort of dogs that initially presented to the emergency or neurology services for clinical signs potentially compatible with intracranial disease (i.e., cardiogenic syncope) but that were ultimately diagnosed with non-neurological disease were also included. Healthy dogs and those with non-neurological illness were recruited using record search, caregiver contact, and questionnaire administration methods as described for dogs with intracranial disease. In instances which the caregivers did not complete the online questionnaire prior to their scheduled appointment, they were given an additional opportunity to complete a written version the day of their visit. To assess the test-retest reliability of the items, caregivers of dogs returning for re-evaluation at least 3 weeks after completion of the first questionnaire were again asked to complete the on-line survey and restrict their answers to events in the 7 days that preceded their receipt of the survey. Approval for the study was granted by the institutional hospital review board.

### Statistical Analysis

For item retention, a principal component analysis (PCA) was performed on responses to the CanBrainQOL-39 questionnaire using ones as prior communality estimates. The principal axis method was used to extract the components, and this was followed by a varimax rotation. A 6-component PCA model that explained 67% of data variation was used to select items for the final questionnaire. Within each of the 3 domains, internal reliability was assessed using the Chronbach's alpha coefficient. Scores for the entire questionnaire and for each of the domains were computed by summing the individual item responses. Normal probability plots showed that total CanBrainQOL-24 score (total instrument and for each of the domains), KPS score, and VAS score followed an approximately normal distribution.

To assess if the final questionnaire was able to discriminate between: (1) healthy and diseased dogs, and (2) dogs with intracranial disease and dogs with non-neurological illness, the means scores for the two comparator groups were evaluated using 2-sample *t*-tests. To assess if the instrument was able to discriminate among the various etiologies of intracranial disease, the groups were compared using a one-way analysis of variance followed by Tukey's procedure for multiple comparisons. Test-retest reliability was evaluated using the intraclass correlation coefficient. Associations between owner reported VAS and total CanBrainQOL-24 scores and clinician derived functional indices (MGCS and KPS) were assessed using scatter plots followed by correlation analysis. The association between CanBrainQOL-24/VAS and CPS (3 levels) was assessed using one-way analysis of variance followed Tukey's procedure for multiple comparisons. Statistical significance was set to *p* < 0.05. All analyses were performed using SAS version 9.4 (Cary, NC, USA).

## Results

A total of 191 CanBrainQOL-39 questionnaires were distributed, to which 140/191 (73%) unique respondents provided replies. Ultimately, data from 128 dogs were included in the study, as 12 surveys were excluded from analysis because of incomplete responses in written versions of the survey (11/12), or the caregiver completed the questionnaire for a dog in the household that was not the intended target of the survey (1/12). Questionnaires were completed for 56 dogs diagnosed with brain diseases, 52 healthy dogs, and 20 dogs with non-neurological illnesses (Table 1). Diagnoses in the intracranial disease group included congenital hydrocephalus (4/56), brain tumors (20/56), IE (8/56), MUE (12/56), ischemic stroke (5/56), and TBI (7/56). Among dogs with intracranial diseases, 55% (31/56) of dogs experienced seizures including the eight dogs with IE and another 23 dogs with concurrent structural epilepsy (2/4 with congenital hydrocephalus, 14/20 with brain tumors, 5/12 with MUE, 1/5 with stroke, and 1/7 with TBI). Diagnoses in the non-neurological disease group included: arrhythmogenic right ventricular cardiomyopathy (3/20), primary immune-mediated polyarthritis (3/20), sudden acquired retinal degeneration syndrome (2/20), pulmonary hypertension and thromboembolism (2/20), immune-mediated hemolytic anemia (2/20), right atrial hemangiosarcoma (2/20), high-grade atrioventricular blockade (2/20), Stage 3b T-cell lymphoma (1/20), hepatozoonosis (1/20), gastrointestinal stromal tumor (1/20), and spontaneous pneumothorax (1/20). Based on the 94 surveys that were submitted electronically, the mean time for a caregiver to complete the survey was 14.8 ± 3 min.

**Table 1:** Descriptive demographic statistics and group assignments of dogs evaluated with the CanBrainQOL-39 survey.

	**Group**	***N***	**Breed**	Age^*^	**Sex**				BW^*^ (kg)
					**F**	**FS**	**M**	**MN**	
	Healthy	52	Mixed (15), Labrador (5), Am. Staff Terrier (5), Aus. Shepherd (4), Beagle (4), Boston terrier (3), Boxer (3), Eng. Bulldog (2), Golden Ret. (2), Great Dane (2), Pug, Rat terrier, Siberian Husky, Silkie terrier, Spitz, Weimeraner, Yorkshire terrier	5.5 (1-12)	1	28	0	23	19 (3-52)
Other Disease	Non-neurologic diseases	20	Mixed (4), Min. Poodle (3), Boxer (3), Min. Schnauzer (2), German shepherd (2), Beagle, Ches. Bay Ret., Collie, Golden Ret., Labrador, Maltese	7.5 (4-11)	1	8	2	9	29 (3-43)
Intra-cranial disease	Congenital hydrocephalus	4	Chihuahua (2), Eng. Bulldog, Yorkshire terrier	1.8 (1-4)	0	2	0	2	3 (2-24)
	Brain tumor	20	Boxer (4), Boston terrier (2), Golden Ret. (2), Mixed (2), Labrador (2), Am. Bulldog, Boykin spaniel, Bullmastiff, Collie, Fox terrier, Min. Schnauzer, Welsh corgi	8 (5-13)	0	12	1	7	23 (8-42)
	Idiopathic/ Genetic epilepsy	8	Mixed (2), Aus. Shepherd, German Shepherd, Golden Ret., Labrador, Poodle, Weimeraner	3.5 (2-5)	0	4	0	4	25 (8-41)
	MUE	12	Pug (3), Mixed (2), Chihuahua (2), Maltese (2), Poodle, Rat Terrier, Yorkshire terrier	4 (2-6)	1	6	0	5	7 (2-13)
	Stroke	5	Chow, Greyhound, Mixed, Rat Terrier, WHW Terrier	8 (6-12)	0	2	0	3	7 (5-28)
	Traumatic brain injury	7	Mixed (2), Labrador (2), German shepherd, Rottweiler, Shetland Sheepdog	4 (1-8)	0	3	0	4	19 (8-40)
	Totals	128		6 (1-13)	3	65	3	57	24 (2-52)

* *Data presented as median (range). BW, body weight; F, intact female; FS, spayed female; M, intact male; MN, neutered male*.

Overall, 63% (80/128) of caregivers responded affirmatively to close-ended questions in the third section of the questionnaire. The frequencies of respondents that provided at least one affirmation to a closed-ended question included 53/56 caregivers of dogs with intracranial disease, 17/20 caregivers of dogs with non-neurological illness, and 10/52 caregivers of healthy dogs. Among affirmative responses, 92% (73/80) were related to responses to survey items that were attributable to treatment(s) administered to their dog. Open-ended responses indicated that therapies prescribed to dogs in this population had the potential to influence every item and domain in the survey, but perceived effects of treatment most frequently (75%; 60/80) involved the emotional well-being and human-animal interaction domain, and therapeutic effect attributions influenced items in at least 2 domains in all dogs. Perceived beneficial effects of treatment were specifically described by caregivers in 45% (36/80) of surveys, and these were most frequently (67%; 24/36) stated to positively influence the emotional well-being and human-animal interaction domain. Adverse effects of treatment were specifically identified in open-ended responses from 40% (32/80) of caregivers that completed this portion of the survey, and these were universally (32/32) perceived to negatively influence the physical domain, as well as the emotional well-being (21/32), and brain-specific (16/32) domains. No attempt was made to further analyze any possible treatment associations, as most dogs received multiple and highly variable therapies, some caregivers did not specify or clarify which treatment they were attributing their responses to, and other responses were ambiguous.

Following PCA, the final questionnaire, termed the CanBrainQOL-24, was reduced to 24 items with minimum Cronbach's alpha values of 0.70 within the three domains (Table 2).

**Table 2:** Final CanBrainQOL-24 instrument.

		**Item** **Likert Rating Scale**	**Cronbach's** **Alpha (range)**
**Item**	**Domain-physical well being**		
1	Does your dog appear sick or ill?	(1-5) No, a little bit, somewhat, quite a bit, very much	0.81 (0.79–0.83)
2	Do you think your dog is in pain?		
3	Does your dog have any side effects of treatment?		
4	Is your dog eating more or wanting to eat more?		
5	Is your dog drinking more or wanting to drink more?		
6	Have your dog's housetraining habits changed?		
7	Has your dog gained weight?		
8	Has your dog lost weight?		
9	How often is your dog in pain?		
10	Is your dog's appetite or thirst decreased?	(1-5)Never, Rarely, Same as before, Frequently, Always	
11	Does your dog have problems with mobility (difficulty getting up, walking, running, or posturing to defecate or urinate)?		
12	Does your dog have bowel problems (having diarrhea, constipation, or accidents in the house)?		
13	Does your dog have bladder problems (urinating more or less frequently, or having accidents in house)?		
14	Does your dog sleep more than before?		
15	Does your dog get tired easily?		
**Item**	**Domain-social interaction/companionship**		
16	Is your dog attentive to his or her caregiver(s)?	(1-5) More than before, same as before, less than before, rarely, never	0.77 (0.73–0.79)
17	Does your dog respond to its caregiver(s) affection?		
18	Does your dog express interest or happiness?		
19	Has your dog's behavior or personality changed?	(1-5) No, a little bit, somewhat, quite a bit, very much	
**Item**	**Domain-brain-specific concerns**		
20	Does your dog have problems with his or her vision/eyes?	(1-5) No, a little bit, somewhat, quite a bit, very much	0.75 (0.66–0.83)
21	Does your dog have balance problems?		
22	Does your dog appear uncoordinated or clumsy?		
23	Does your dog have weakness in its front or back legs?		
24	Does your dog have seizures, convulsions, or fits?		

*Caregivers completed the questionnaire in the context of the degree to which clinical signs or treatment of intracranial disease affected their dog's QOL for the 7 days preceding their completion of the survey*.

Healthy dogs had significantly lower CanBrainQOL-24 scores than dogs with intracranial or other diseases (Table 3). Total CanBrainQOL-24 scores were not significantly different between clinically ill dogs with or without intracranial disease. Dogs with non-neurological illness had significantly higher physical domain scores than dogs with intracranial disease, and dogs with intracranial disease had significantly higher brain domain scores than dogs with non-neurological illnesses (Table 3). Total and domain specific CanBrainQOL-24 scores were not different between dogs with different etiologies of intracranial disease.

**Table 3:** Discriminatory analyses of the CanBrainQOL-24 instrument.

**Instrument component**	**Comparisons**	**Category**	***N***	**QOL score** **Mean (± SD)**	***P*-value**
CanBrainQOL-24 total	Healthy vs. disease	Healthy	52	29.4 (2.5)	<0.0001^*^
		Disease	76	44.2 (9.5)	
CanBrainQOL-24 total	Other disease vs. intracranial disease (ICD)	Other	20	45.0 (12.9)	0.79
		ICD	56	44.0 (8.9)	
•Physical domain	Other disease vs. intracranial disease	Other	20	30.8 (10.4)	0.04^*^
		ICD	56	25.6 (5.6)	
•Social domain	Other disease vs. Intracranial disease	Other	20	8.6 (2.1)	0.76
		ICD	56	8.9 (2.0)	
•Brain domain	Other disease vs. intracranial disease	Other	20	5.6 (1.2)	0.002^*^
		ICD	56	9.5 (3.4)	
CanBrainQOL-24 total	Intracranial disease etiology	CH	4	44.1 (6.2)	0.47
		Tumor	20	43.3 (10.0)	
		MUE	12	48.9 (8.1)	
		IE	8	41.6 (10.7)	
		Stroke	5	43.0 (3.7)	
		TBI	7	42.3 (4.3)	
•Physical domain	Intracranial Disease Etiology	CH	4	24.5 (4.2)	0.43
		Tumor	20	24.9 (5.5)	
		MUE	12	28.9 (5.3)	
		IE	8	24.6 (8.1)	
		Stroke	5	24.6 (1.9)	
		TBI	7	24.8 (1.7)	
•Social domain	Intracranial disease etiology	CH	4	9.3 (2.5)	0.92
		Tumor	20	9.1 (2.7)	
		MUE	12	9.1 (1.6)	
		IE	8	8.8 (2.0)	
		Stroke	5	8.2 (1.3)	
		TBI	7	8.5 (0.6)	
•Brain domain	Intracranial disease etiology	CH	4	9.8 (3.1)	0.58
		Tumor	20	9.3 (4.2)	
		MUE	12	10.9 (3.8)	
		IE	8	8.3 (1.4)	
		Stroke	5	10.2 (2.9)	
		TBI	7	9.0 (2.4)	

* *Significant value (P < 0.05). CH, congenital hydrocephalus; IE, idiopathic/genetic epilepsy; MUE, meningoencephalitis of unknown etiology; TBI, traumatic brain injury*.

A significant positive correlation was observed between total CanBrainQOL-24 and owner reported VAS scores (*R*^2^ = 0.9, 95% CI: 0.83–0.94, *p* < 0.001), and significant negative correlations observed between CanBrainQOL-24 and KPS (*R*^2^ = −0.87, 95% CI: −0.92 to −0.78, *p* < 0.001), CanBrainQOL-24 and MGCS (*R*^2^ = −0.47, 95% CI: −0.65 to −0.25, *p* < 0.001), VAS and MGCS (*R*^2^ = −0.51, 95% CI: −0.67 to −0.30, *p* < 0.001), and VAS and KPS (*R*^2^ = −0.82, 95% CI: −0.89 to −0.78, *p* < 0.001). All associations between owner reported VAS and CanBrainQOL-24 scores and clinician assigned CPS were also significant (Table 4). A total of 60 surveys were redistributed to caregivers of healthy (*n* = 30) and diseased dogs (*n* = 30) to evaluate test-retest reliability, to which 35/60 (58%) responses were received from caregivers of 21 dogs with intracranial disease and 14 healthy dogs (Table 5). The intraclass correlation coefficients (ICC) evaluating the test-retest reliability were as follows: total CanBrainQOL-24 (0.41), physical domain (0.17), social domain (0.35), and brain domain (0.57).

**Table 4:** Associations between canine Chronic Pain Scores and owner reported total CanBrainQOL-24 and Visual Analog (VAS) Quality of Life Scores.

**One way analyses**				**Two way analyses**			
**Chronic pain score**	**VAS estimate**	**Standard error**	***P*-value**	**Chronic pain score comparison**	**VAS difference estimate**	**Standard error**	***P*-value**
0	21.7	1.87	<0.0001	0 vs. 1	−10.32	3.70	0.02
1	32.0	3.19	<0.0001	0 vs. 2	−23.89	4.89	<0.0001
2	45.7	4.52	<0.0001	1 vs. 2	−13.57	5.53	0.04
	**CanBrainQOL-24 estimate**				**CanBrainQOL-24 difference estimate**		
0	37.5	1.33	<0.0001	0 vs. 1	−7.89	2.63	0.01
1	45.6	2.27	<0.0001	0 vs. 2	−18.40	3.47	<0.0001
2	55.9	3.21	<0.0001	1 vs. 2	−10.50	3.93	0.03

*All P-values significant (P < 0.05)*.

**Table 5:** Group assignment, selected descriptive demographic statistics, and outcome measures of dogs evaluated in test-retest reliability of CanBrainQOL-24.

	**Group**	***N***	Age^*^	Δ (±) VAS between surveys^*^	Δ (±) KPS between surveys^*^	Δ (±) CanBrainQOL-24 between surveys^*^
	Healthy	14	9 (4-12)	4 (0–5)	0	3 (0–4)
Intra-cranial disease	Brain tumor	8	8 (8-13)	18 (3-48)	15 (0–35)	17 (9-42)
	Idiopathic epilepsy	3	3.5 (3-4)	17.5 (6-29)	10 (10-20)	12 (5-19)
	MUE	7	4 (3-5)	13 (2-23)	12 (0–20)	16 (8-28)
	Traumatic brain injury	3	4 (3-6)	21 (13-52)	20 (10-40)	34 (16-44)

* *Data presented as median (range). KPS, Karnofsky performance score; VAS, Visual analog scale QOL score*.

## Discussion

Results of this study indicated that the CanBrainQOL-24 survey can be used to assess HRQOL in dogs with intracranial disease. Principal component analysis allowed for a reduction in the number of items without significantly affecting the descriptive value of the survey. Cronbach's alpha values indicated acceptable internal consistency of item clustering within the three domains, and that these domains can be reliably evaluated with the CanBrainQOL-24. The CanBrainQOL-24 clearly discriminated healthy from diseased dogs, with total CanBrainQOL-24 scores being significantly higher in dogs with clinical illnesses. There was also a significant and strong positive correlation between both caregiver reported assessments, the VAS and total CanBrainQOL-24 scores.

VAS and CanBrainQOL-24 scores were also shown to significantly correlate to clinician derived health surrogates including the CPS, KPS, and MGCS, and that the directionality of these relationships was in inherent agreement with expected clinical outcomes. For example, VAS and CanBrainQOL-24 scores were significantly but negatively correlated with KPS, indicating that proxy reported HRQOL was worse in those animals with evidence of more severe clinical dysfunction. Additionally, there were significant associations between VAS, CanBrainQOL-24, and all CPS scores, supporting results of previous investigations indicating that pain is an important factor contributing to HRQOL in animals (10, 11).

Collectively, the CPS, KPS, and MGCS provide information about the severity of disease through the assessment of physiological variables and interrogation of neurological functions, while the VAS and CanBrainQOL-24 score describe how a proxy perceives the disease's impact on the dog's QOL (24–26). Our data indicate that an owner-reported HRQOL surrogate provided information that complements health data derived from the history and clinical examination. Our study and others also suggest that a simple and direct QOL question, such as the VAS, may also provide an accurate representation of the animal's QOL, and that QOL assessments of various complexity and formats can be useful for the evaluation of patient-centered outcomes in all facets of veterinary practice (27). However, for use in clinical trials, it has been recommended that disease-specific instruments be used when attempting to evaluate treatment effects (28). As indicated by caregiver open-ended survey responses in this study, prescribed therapies were perceived to positively and negatively influence QOL with nearly equal frequency, and these effects encompassed all evaluated domains. Correlations between caregiver reported and clinician derived measures provide a valuable contextual framework for discussions between health care providers and animal caregivers outlining goals and expectations associated with treatment of intracranial diseases, and emphasize the shared objective of improving the welfare of the animal.

Although there was statistically significant alignment between caregiver reported and clinician determined measures of health in this study, a notable area of divergence between clinician assessments and owner perception was observed in dogs with genetic or structural epilepsy (5, 14). Discordance between VAS/Can-BrainQOL-24 and CPS, KPS, and MCGS scores frequently manifested as the clinician overestimating the dog's general welfare compared to caregiver reports. Although the dynamic and multidimensional impacts of epilepsy on both caregivers and animals QOL are well described, there is no reference standard that has been validated to assess QOL in dogs with epilepsy, and at the time the CanBrainQOL-39 was developed there was no survey instrument validated for use in dogs with epilepsy (16). Importantly, none of the measures of clinical health assigned by veterinarians in this study were specifically designed to assess the effects of epilepsy on patient performance status nor allow for objective assessment of social interaction or companionship QOL domain (24–26). Given the high frequency of perceived positive and negative effects of treatments on the social interaction and emotional well-being domains observed in this study, our results indicate that these instruments are insensitive to the detection of potentially impactful QOL concerns observed by caregivers of dogs with intracranial disease. Thus, future studies should consider factor loading to determine the impacts of individual factors on QOL, or to conduct seizure outcome assessments that are focused and distinct from other QOL questionnaires (12, 29).

Our results indicate that the CanBrainQOL-24 instrument was unable to differentiate between dogs with intracranial disease and those with non-neurologic illnesses, and that CanBrainQOL-24 scores did not differ significantly between the various etiologies of intracranial disease included in the study. However, when compared to dogs with non-neurological disorders, dogs with intracranial disease had significantly higher brain domain scores and significantly lower physical domain scores. These observations have prompted us to consider inclusion of domain-weighted QOL scores in future studies. Domain-weighting provides an opportunity for caregivers to identify items they perceive have the greatest influence on QOL in their dogs, and differentially grade the importance of these items (13). Knowledge of the specific aspects of QOL that are particularly valued by the caregiver would also allow clinicians to develop and communicate individualized treatment plans more effectively.

In this study, the intraclass correlation coefficients for each of the domains were low, indicating that the test-retest reliability of the survey was poor to moderate. However, these results should be interpreted cautiously. The dogs evaluated with intracranial disease experienced considerable changes in their KPS, VAS, and CanBrainQOL-24 scores between initial and subsequent evaluations. Similar to what has been observed in dogs with spinal cord injury and brain tumors (13, 15), our results indicate that canine intracranial disease and its treatment are dynamic processes, and that evolutions in caregiver attitudes toward QOL over the disease course could be an additional source of the observed variation. Further research is needed to evaluate the test-retest reliability of the CanBrainQOL-24 survey in clinically stable populations.

## Conclusions

Our findings indicate that the CanBrainQOL-24 instrument provides core items useful for the assessment of HRQOL in dogs with intracranial disease. However, future investigations are required to refine intrinsic assessment items within the domains, further define the reliability of the questionnaire, and to evaluate its validity when administered to larger populations with more diverse etiologies of intracranial disease.

## Data Availability Statement

The raw data supporting the conclusions of this article will be made available by the authors, without undue reservation.

## Ethics Statement

The animal study was reviewed and approved by the Virginia Tech Institutional Animal Care and Animal Use Committee and the Veterinary Teaching Hospital Review Board (08-48, 12-014, 17-203). Written informed consent was obtained from the owners for the participation of their animals in this study.

## Author Contributions

RW drafted the manuscript. MS, MQ, TP, and JR participated in survey generation and data collection and management. RW, TP, SW, and JR contributed to data analysis. SW and JR designed the study. RW and JR critically revised the manuscript. All authors read and approved the final manuscript.

## Conflict of Interest

The authors declare that the research was conducted in the absence of any commercial or financial relationships that could be construed as a potential conflict of interest. The handling editor declared a past co-authorship with the authors TP and JR.
