# Supplementary material for: Development and Evaluation of a Caregiver Reported Quality of Life Assessment Instrument in Dogs With Intracranial Disease
Source: Front Vet Sci. 2020 Aug 18;7:537. doi: 10.3389/fvets.2020.00537 (PMC7461854; doi:10.3389/fvets.2020.00537)
Supplement: Supplementary file 1 [file Table_1.DOCX]

**Supplemental Data**

**Table S1- CanBrainQOL-39 Survey Instrument, Section 2 Items**

|  |  | **Item**  **Likert Rating Scale** |
| --- | --- | --- |
| **Item** | **Domain-Physical Well Being** | |
| 1 | Is your dog lethargic? | (1-5)  No, A little bit, Somewhat, Quite a bit, Very much |
| 2 | Does your dog appear sick or ill? |  |
| 3 | Does your dog’s physical condition interfere with his or her activity or lifestyle? |  |
| 4 | Has your dog’s sleeping habits changed? |  |
| 5 | Have your dog’s hygienic habits changed (does it groom itself)? |  |
| 6 | Do you think your dog is in pain? |  |
| 7 | Does your dog have any side effects of treatment? |  |
| 8 | Is your dog eating more or wanting to eat more? |  |
| 9 | Is your dog drinking more or wanting to drink more? |  |
| 10 | Have your dog’s housetraining habits changed? |  |
| 11 | Has your dog gained weight? |  |
| 12 | Has your dog lost weight? |  |
| 13 | How often is your dog in pain? | (1-5)  Never, Rarely, Same as before, Frequently, Always |
| 14 | Does your dog vomit? |  |
| 15 | Does your dog have problems with mobility (difficulty getting up, walking, running, or posturing to defecate or urinate)? |  |
| 16 | Does your dog have bowel problems (having diarrhea, constipation, or accidents in the house)? |  |
| 17 | Does your dog have bladder problems (urinating more or less frequently, having accidents in house)? |  |
| 18 | Does your dog have difficulty breathing? |  |
| 19 | Is your dog’s appetite or thirst decreased? |  |
| 20 | Does your dog sleep more than before? |  |
| 21 | Does your dog appear restless or anxious? |  |
| 22 | Does your dog get tired easily? |  |
| **Item** | **Domain- Emotional Well-Being/Human-Animal Interaction** | |
| 23 | Is your dog attentive to his or her caregiver(s)? | (1-5)  More than before, Same as before, Less than before, Rarely, Never |
| 24 | Does your dog respond to its caregiver(s) affection? |  |
| 25 | Is your dog responsive to or interactive in his or her environment (family, toys, etc.)? |  |
| 26 | Does your dog express interest or happiness? |  |
| 27 | Does your dog engage in activities he or she usually enjoys? |  |
| 28 | Has your dog’s behavior changed? | (1-5)  No, A little bit, Somewhat, Quite a bit, Very much |
| 29 | Has your dog’s mood changed? |  |
| 30 | Does your dog require your help with daily functions? |  |
| **Item** | **Domain- Brain Specific Items** | |
| 31 | Does your dog have problems with vision/eyes? | (1-5)  No, A little bit, Somewhat, Quite a bit, Very much |
| 32 | Does your dog have problems hearing? |  |
| 33 | Does your dog have seizures, convulsions, or fits? |  |
| 34 | Does your dog have balance problems? |  |
| 35 | Does your dog appear uncoordinated or clumsy? |  |
| 36 | Does your dog have weakness in its front or back legs? |  |
| 37 | Does your dog display repetitive behaviors (circling, pacing, head pressing, vocalizing) |  |
| 38 | Does your dog have tremors or spasms? |  |
| 39 | Has your dog’s personality changed? |  |

Caregivers completed the questionnaire in the context of the degree to which clinical signs or treatment of intracranial disease affected their dog’s QOL for the 7 days preceding their completion of the survey.
